# Role of casein kinase 1 in the glucose sensor-mediated signaling pathway in yeast

**DOI:** 10.1186/1471-2121-11-17

**Published:** 2010-03-07

**Authors:** Satish Pasula, Samujjwal Chakraborty, Jae H Choi, Jeong-Ho Kim

**Affiliations:** 1 The Mississippi Functional Genomics Network, Department of Biological Sciences, The University of Southern Mississippi, 118 College Dr, Hattiesburg, MS 39406, USA; 2 University of Illinois at Chicago College of Pharmacy, Department of Medicinal Chemistry and Pharmacognosy, Rockford Regional Program, 1601 Parkview Avenue, Rockford, IL 61107, USA; 3 Oklahoma Medical Research Foundation, 825 N.E. 13th Street, Oklahoma City, Oklahoma 73104, USA

## Abstract

**Background:**

In yeast, glucose-dependent degradation of the Mth1 protein, a corepressor of the glucose transporter gene (*HXT*) repressor Rgt1, is a crucial event enabling expression of several *HXT*. This event occurs through a signaling pathway that involves the Rgt2 and Snf3 glucose sensors and yeast casein kinase 1 and 2 (Yck1/2). In this study, we examined whether the glucose sensors directly couple with Yck1/2 to convert glucose binding into an intracellular signal that leads to the degradation of Mth1.

**Results:**

High levels of glucose induce degradation of Mth1 through the Rgt2/Snf3 glucose signaling pathway. Fluorescence microscopy analysis indicates that, under glucose-limited conditions, GFP-Mth1 is localized in the nucleus and does not shuttle between the nucleus and cytoplasm. If glucose-induced degradation is prevented due to disruption of the Rgt2/Snf3 pathway, GFP-Mth1 accumulates in the nucleus. When engineered to be localized to the cytoplasm, GFP-Mth1 is degraded regardless of the presence of glucose or the glucose sensors. In addition, removal of Grr1 from the nucleus prevents degradation of GFP-Mth1. These results suggest that glucose-induced, glucose sensor-dependent Mth1 degradation occurs in the nucleus. We also show that, like Yck2, Yck1 is localized to the plasma membrane via C-terminal palmitoylation mediated by the palmitoyl transferase Akr1. However, glucose-dependent degradation of Mth1 is not impaired in the absence of Akr1, suggesting that a direct interaction between the glucose sensors and Yck1/2 is not required for Mth1 degradation.

**Conclusion:**

Glucose-induced, glucose sensor-regulated degradation of Mth1 occurs in the nucleus and does not require direct interaction of the glucose sensors with Yck1/2.

## Background

In the budding yeast *Saccharomyces cerevisiae*, glucose stimulates its transport across the plasma membrane by inducing expression of several *HXT *[1-3]. Under glucose-limited conditions, the transcriptional repressor Rgt1 binds to the *HXT *promoters and recruits general corepressors Ssn6 and Tup1 [4-7]. Rgt1 does this in conjunction with its corepressor Mth1, which physically interacts with Rgt1 [8-10]. Therefore, it has been proposed that Rgt1 forms a repression complex with Mth1, Ssn6, and Tup1 on the *HXT *promoters, inhibiting transcription [6]. Glucose appears to prevent formation of this protein complex by causing degradation of Mth1, resulting in release of Rgt1 from *HXT *promoters, thereby inducing expression of *HXT *[6,11-14].

The glucose signal that leads to degradation of Mth1 is generated by the plasma-membrane spanning glucose sensors Rgt2 and Snf3. Signal generation is a receptor-mediated process and does not require glucose metabolism. This idea is supported by evidence that dominant mutations exist in the glucose sensor genes that lock the sensor proteins in glucose-bound conformations, generating a constitutive signal [15,16]. Indeed, Mth1 is constitutively degraded in cells expressing the active glucose sensor mutants [17]. Subsequent studies have shown that the plasma membrane-tethered casein kinases Yck1/2 phosphorylate Mth1, triggering its ubiquitination and subsequent degradation [18]. It has also been shown by yeast-two-hybrid assay that Mth1 interacts with the C-terminal tails of the glucose sensors, suggesting that Mth1 is recruited to the plasma membrane [19-21]. These observations have led to the current view of glucose-induced *HXT *expression. Upon glucose binding, the glucose sensors are converted from inactive to active forms through a conformational change, activating Yck1/2 in their vicinity. Mth1, recruited by the glucose sensors to the plasma membrane, is phosphorylated by Yck1/2 and, subsequently, ubiquitinated by SCF^Grr1^. Finally, the ubiquitinated Mth1 is targeted for degradation by the 26S proteasome [12-14,17].

However, this hypothesis is mainly based on the following assumptions: (1) Mth1 is excluded from the nucleus upon glucose addition and recruited to the plasma membrane by any means, and (2) Yck1/2 are activated through a direct interaction with the glucose sensors. In this study, we specifically tested these assumptions and provide evidence that Yck1/2 do not directly couple to the glucose sensors during transmission of the glucose signal from the plasma membrane to the nucleus. A possible mechanism for how the glucose sensors and Yck1/2 collaborate to degrade Mth1 is discussed.

## Results

### Subcellular localization of Mth1 is not regulated

**Figure 1:**
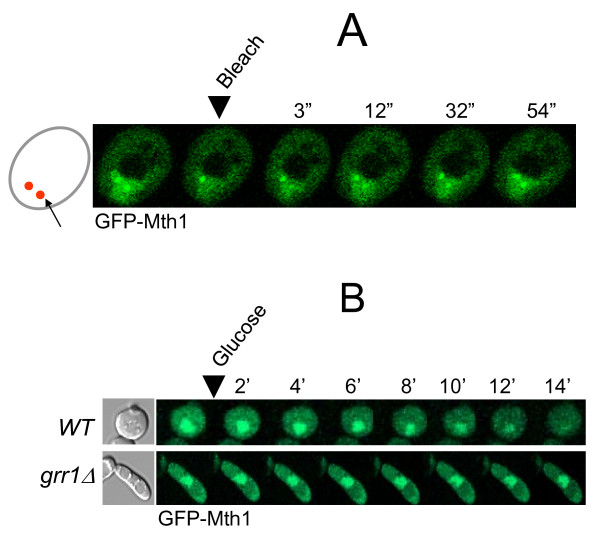
**Mth1 does not shuttle between the nucleus and cytoplasm**. (A) Images of GFP-Mth1 expressing yeast cells grown in galactose (2%) before and after photobleaching in the indicated region. One of two foci (arrow) was bleached and then recovery of the fluorescence in the bleached zone was monitored. Time is in seconds after photobleaching. (B) Time-lapse observation of Mth1 degradation in high-glucose medium (4%). Numbers indicate time in minutes. Yeast cells, wild-type (*WT*; BY4742) and *grr1 *mutant (*grr1Δ*; YM4783), expressing GFP-Mth1 were observed under the Zeiss LSM 510 META Confocal Laser Scanning Microscope (CLSM).

We have previously reported that GFP-Mth1 is mainly nuclear in glucose-depleted medium and is rapidly degraded upon glucose addition [14]. To gain more insights into glucose-dependent degradation of Mth1, we first examined subcellular localization of GFP-Mth1 in cells grown under conditions where *HXT *expression is repressed (2% galactose) or induced (4% glucose) (Figure 1). Because glucose also regulates expression of *MTH1 *[22], *GFP-MTH1 *was expressed from the *MET25 *promoter, which is not regulated by glucose, in a low copy centromeric plasmid [14]. Fluorescence microscopy analysis demonstrates that GFP-Mth1 is localized primarily to two specific foci within the nucleus and that ~50% of fluorescence is recovered within 30-40 seconds after photobleaching (Figure 1A). These results suggest that, under glucose-limited conditions, Mth1 predominantly accumulates in the nucleus but does not shuttle between the nucleus and cytoplasm. We also monitored glucose-induced degradation of Mth1 in a time-lapse manner and observed that more than half of GFP-Mth1 is degraded within 5-10 min after glucose addition (Figure 1B), which is consistent with the Western blot analysis obtained previously [14].

**Figure 2:**
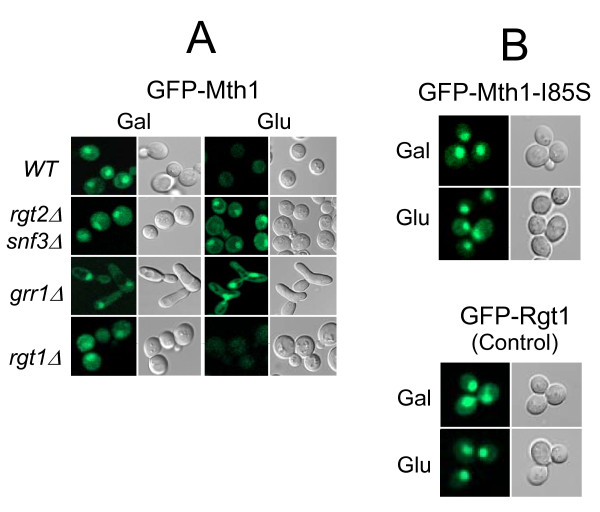
**Neither glucose nor the glucose sensors influence the subcellular localization of Mth1**. (A) Yeast cells of the indicated genotype expressing GFP-Mth1 were grown to mid-log phase in a selective medium containing 2% galactose. Aliquots were then transferred to 2% galactose medium (Gal) or 4% glucose medium (Glu) and incubated for 60 min. Subcellular localization of GFP-Mth1 was analyzed by fluorescence microscopy. Yeast cells used were: *WT *(BY4742), *rgt2Δsnf3Δ *(YM6370), *grr1Δ *(YM4783), and *rgt1Δ *(YM4509). (B) Localization of a nondegradable form of Mth1 (Mth1-I85S) in wild-type cells (BY4742). (C) GFP-Rgt1 localization as a positive control.

To determine whether Mth1 is excluded from the nucleus in response to glucose, we determined in which cellular compartment Mth1 is localized when expressed under high glucose conditions. Toward this aim, we examined subcellular localization of GFP-Mth1 in the *rgt2snf3 *and *grr1 *mutants, where glucose-dependent degradation of Mth1 is severely impaired [14]. As shown in Figure 2A, the GFP-Mth1 proteins accumulate in the nucleus of the mutants grown in high-glucose medium. To further test a possibility that the glucose sensors regulate nuclear exclusion of Mth1, we determined subcellular localization of a nondegradable form of Mth1 in wild-type cells, where the glucose sensors are active. The dominant *HTR1-23 *mutation in *MTH1 *(I85S) [23] converts Mth1 into a degradation-resistant form [14]. Our results indicate that the mutant Mth1 proteins (GFP-Mth1-I85S) are not degraded, as expected, but accumulate in the nucleus in high-glucose medium (Figure 2B). We also observed that subcellular localization and degradation of Mth1 are not influenced by the absence of Rgt1, a downstream target of the Rgt2/Snf3-mediated signaling pathway [22]. Therefore, it is likely that neither glucose nor the glucose sensors control nuclear export of Mth1.

### Glucose-dependent degradation of Mth1 occurs in the nucleus

**Figure 3:**
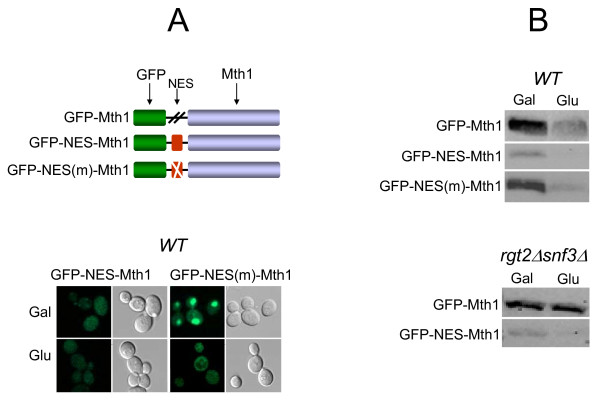
**Glucose-dependent degradation of Mth1 takes place in the nucleus**. Synthetic oligonucleotides encoding the nuclear export sequence (NES) and its mutant peptide (NES (m)) were inserted between the *GFP *and *MTH1 *genes in the *GFP-MTH1 *construct. Yeast cells (*mth1Δ*, YM6266) expressing GFP-Mth1 and its derivatives were grown as described in Figure 2. Subcellular localization and levels of GFP-Mth1 were analyzed by fluorescence microscopy using CLSM (A) and Western blotting using anti-GFP antibody (B), respectively.

To more directly assess whether Mth1 degradation occurs in the nucleus or cytoplasm, we examined degradation of GFP-Mth1 proteins engineered to have cytoplasmic localization. To do so, GFP-Mth1 was tagged with the nuclear export signal (NES) of the yeast PKIα (GFP-NES-Mth1) [24]. To test the functionality of the NES motif, we constructed plasmid expressing GFP-NES(m)-Mth1 containing a single amino acid substitution within the motif. The levels and subcellular localization of the resulting fusion proteins were determined by Western blot and fluorescence microscopy analysis, respectively (Figure 3). We observed that GFP-NES-Mth1 is excluded from the nucleus, as expected (Figure 3A, bottom panel), but significantly degraded regardless of the presence of glucose or the glucose sensors (Figure 3B). In contrast, GFP-Mth1 with a nonfunctional NES motif (GFP-NES(m)-Mth1) behaves like GFP-Mth1; it accumulates in the nucleus in the absence of glucose (2% Gal) but is degraded when glucose is present (4% Glu), providing evidence that the NES motif used is functional. Therefore, collectively, these results suggest that glucose-dependent degradation of Mth1 occurs in the nucleus.

### Nuclear degradation of Mth1 is Grr1-dependent

**Figure 4:**
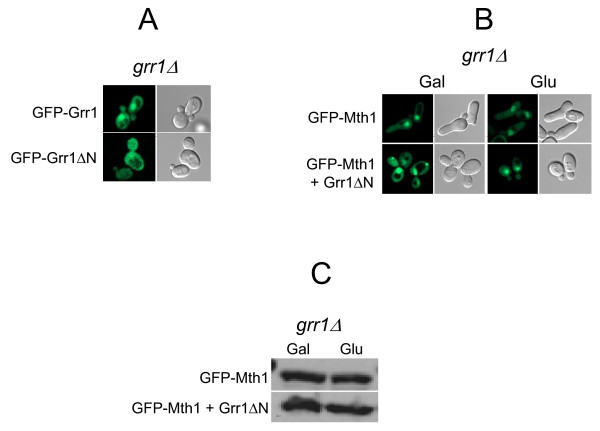
**Glucose-dependent degradation of Mth1 does not occur when Grr1 is removed from the nucleus**. (A) GFP-Grr1 and GFP-Grr1ΔN (lacking the first 280 amino acids of Grr1) were expressed in the *grr1Δ *mutant (YM4783) in high-glucose medium (4%). (B) GFP-Mth1 was expressed alone (top) or coexpressed with Grr1-ΔN (bottom) in the *grr1 *mutant (YM4783). Subcellular localization of GFP-Mth1 and GFP-Grr1ΔN was analyzed by fluorescence microscopy (A and B). (C) GFP-Mth1 was expressed alone (top) or coexpressed with Grr1-ΔN (bottom) in the *grr1 *mutant (YM4783), and levels of GFP-Mth1 were analyzed by Western blotting using anti-GFP antibody. Yeast cells were grown in 2% galactose medium (Gal) or 4% glucose medium (Glu) as described in Figure 2.

Grr1 appears to be present in both the nucleus and cytoplasm, but deletion of the putative NLS in the amino-terminus of Grr1 (Grr1ΔN) causes it to be localized to the cytoplasm [25]. Previous studies have shown that expression of Grr1ΔN is sufficient to restore the morphology of, and to mediate degradation of Gic2 in, the *grr1 *mutant [25,26], suggesting that Grr1ΔN is fully functional in the cytoplasm. To provide more compelling evidence that Grr1-dependent degradation of Mth1 takes place in the nucleus, we examined if GFP-Grr1ΔN (Δ1 280) can mediate Mth1 degradation (Figure 4). Our results show that GFP-Grr1ΔN is predominantly localized to the cytoplasm (Figure 4A) and able to restore the morphology of the *grr1 *mutant (Figure 4B), as reported previously [25,26]. However, expression of Grr1ΔN in the *grr1 *mutant does not cause degradation of GFP-Mth1 (Figure 4C), probably due to translocation of Grr1ΔN from the nucleus to the cytoplasm (Figure 4B). These results suggest that SCF^Grr1^-mediated degradation of Mth1 occurs in the nucleus, not in the cytoplasm.

### Direct interaction between Yck1/2 and the glucose sensors is not required for glucose-dependent degradation of Mth1

**Figure 5:**
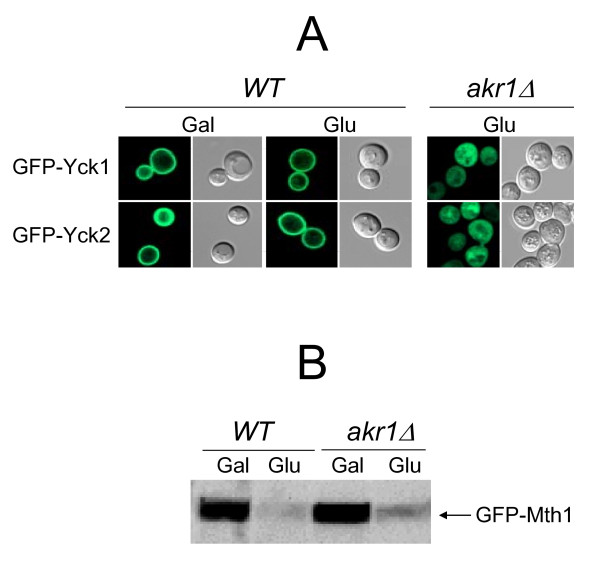
**Direct interaction of Yck1/2 with the glucose sensors is not required for degradation of Mth1**. (A) Subcellular localization of GFP-Yck1 and GFP-Yck2 expressed in wild type (BY4742) and *akr1Δ *mutant (JKY56) strains was examined by fluorescence microscopy. (B) Western blot analysis of GFP-Mth1 in wild-type and *akr1Δ *mutant strains. Yeast cells were grown as described in Figure 2.

Next, we tested whether the glucose sensors are coupled with Yck1/2 in order to transduce glucose binding into an intracellular signaling cascade that leads to Mth1 degradation. Toward this aim, we examined whether Mth1 is degraded when Yck1/2 are mislocalized from the plasma membrane. It has been well described that Yck2 is targeted to the plasma membrane through palmitoylation of the C-terminal Cys-Cys sequence by the palmitoyl transferase Akr1 [27-29]. Confocal microscopy images show that, like GFP-Yck2 [29], GFP-Yck1 is associated with the plasma membrane in wild-type cells, but diffused throughout the entire cell when Akr1 is absent (Figure 5A, *akr1Δ*). Importantly, glucose-induced degradation of Mth1 is not significantly impaired in the absence of Akr1 (Figure 5B). These results suggest that neither localization of Yck1/2 to the plasma membrane nor a direct interaction between Yck1/2 and the glucose sensors is required for degradation of Mth1.

### Four different regions of Mth1 are required for glucose-induced degradation of Mth1

**Figure 6:**
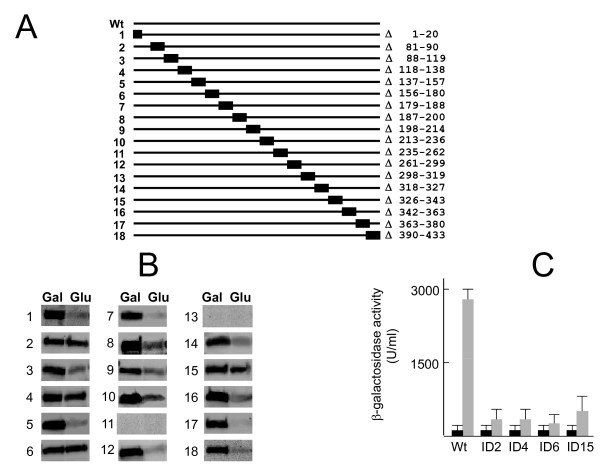
**Identification of the functional regions of Mth1 required for glucose-dependent degradation**. (A) Mth1 deletion mutants were generated to determine the regions responsible for degradation by successive 10-50 amino-acids deletion analysis. In total, 18 deletions were constructed. (B) The mutant Mth1 proteins containing internal deletions were expressed in the *mth1Δ *cells (YM6266) and detected by Western blotting using anti-GFP antibody. (C) Four deletion mutants of Mth1 inhibit glucose-induction of *HXT1 *expression. Four internal deletion constructs without the GFP moiety were coexpressed with the *HXT1*-*lacZ *reporter (pBM2636) in the absence (Gal, black bars) and presence (Glu, gray bars) of glucose.

To identify the regions of Mth1 that are required for its glucose-induced degradation, we constructed a series of successive internal deletions of 10-50 residues covering the entire Mth1 sequence (Figure 6A). The amino acid positions between 20 and 80 of Mth1 were not deleted because this region is not well conserved in the Mth1 orthologs from other yeast species. Most of the deletion mutants of Mth1 protein were detectable by Western blotting, except two mutants that contain Δ235-262 (ID 11) and Δ298-319 (ID 13) (Figure 6B). Our results show that four internal deletions--Δ81-90 (ID 2), Δ118-138 (ID 4), 156-180 (ID 6), and Δ326-343 (ID 15)--render Mth1 resistant to degradation. These Mth1 mutant proteins are constitutively nuclear (data not shown) and significantly inhibit glucose induction of *HXT1 *expression (Figure 6C). Proteasomal degradation of proteins is a multi-step process, involving phosphorylation, ubiquitination, and degradation in the 26S proteasome. Thus, it is likely that these four regions are involved in at least one of these steps.

## Discussion

The yeast glucose sensors convert glucose binding events into an intracellular signal that leads to degradation of Mth1, which is known to require activity of the plasma membrane-tethered Yck1/2 [18]. Because both the glucose sensors and Yck1/2 are associated with the plasma membrane, it has been hypothesized that, upon glucose addition, Yck1/2 are activated through a direct interaction with the glucose sensors and phosphorylate Mth1, triggering its proteasomal degradation.

In this study, we provide several lines of evidence that Mth1 does not shuttle between the nucleus and cytoplasm and is degraded in the nucleus when glucose is present: 1) Mth1 is not excluded from the nucleus in response to glucose (Figure 2); 2) When engineered to be localized to the cytoplasm, Mth1 is degraded in the cytoplasm regardless of the presence of glucose and the glucose sensors (Figure 3); 3) Mth1 is not degraded when Grr1 is removed from the nucleus (Figure 4).

The majority of Yck1/2 targets are plasma membrane proteins. Yck1/2 are responsible for phosphorylating the PEST-like ubiquitination-endocytosis signal of the mating pheromone receptors [30], and the uracil permease Fur4 [31]. These kinases are also known to regulate the activity of the maltose permease Mal61 [32], the multidrug transporter Pdr5 [33], and plasma membrane H^+^-ATPase [34]. Another important target of Yck1/2 is Ptr3, a component of the yeast amino-acid signaling pathway. Extracellular amino acids trigger activation of the Ssy1-Ptr3-Ssy5 (SPS)-amino acid signaling pathway that leads to induction of endoproteolytic processing of Stp1 and Stp2, enabling them to enter the nucleus and induce expression of the amino acid permease genes [35-38]. This processing requires Yck1/2, the novel chymotrypsin like protease Ssy5, and SCF^Grr1 ^[36,37]. Yck1/2 have been shown to phosphorylate the peripheral plasma membrane protein Ptr3 at Thr-525, increasing Ssy5C (C-terminal activity domain)-dependent proteolytic processing of Stp1 and Stp2 [39]. Mth1 is required to form a repression complex with Rgt1 on the *HXT *promoters [6] and appears not to contain endogenous NES-like motifs in its sequence (data not shown). These observations support the idea that Mth1 neither shuttles between the nucleus and cytoplasm nor is excluded from the nucleus. Therefore, it is unlikely that the plasma membrane-tethered Yck1/2 directly phosphorylate the nuclear-localized Mth1.

We also provide evidence that Mth1 degradation is not impaired by the mislocalization of Yck1/2 from the plasma membrane, suggesting that direct interaction between the glucose sensors and Yck1/2 is not required for Mth1 degradation (Figure 5). Although Yck1/2 have been reported to be necessary for Mth1 degradation [18], our results suggest that they do not exert their function through the glucose sensors. It is not known how the glucose binding to the glucose sensors is converted to an intracellular signal that leads to degradation of Mth1. In this regard, we surmise that there is a yet unidentified kinase that receives the glucose signal and converts it into an intracellular signal. In this scenario, the kinase is recruited to the glucose sensors upon glucose addition and phosphorylated by Yck1/2 at the plasma membrane. Finally, the kinase is translocated from the cytoplasm into the nucleus and catalyzes phosphorylation of Mth1, triggering its degradation by the 26S proteasome.

## Conclusions

Glucose-induced, the glucose sensor-regulated Mth1 degradation occurs in the nucleus and requires neither nuclear export of Mth1 nor direct interaction between the glucose sensors and Yck1/2. The glucose sensors transmit their signal across the plasma membrane through a yet unidentified signaling component, not through Yck1/2.

## Methods

### Yeast strains and plasmids

Yeast strains were grown on YPD (1% yeast extract, 2% bacto-peptone, and 4% glucose) or synthetic yeast nitrogen base media (0.17% yeast nitrogen base with 0.5% ammonium sulfate) supplemented with the appropriate amino acids and carbon sources. The *AKR1 *gene was disrupted by homologous recombination using the NatMX cassette [40]. Plasmids that express the mutant Mth1 proteins with internal deletions were constructed by using the gap-repair technique [41]. To construct the *GFP-MTH1 *tagged with either the wild-type (NES, ELALKLAGLDIN) or mutated (NESm, ELALKLAGADIN; L changed to A (Underlined)) leucine-rich nuclear export sequences of the yeast PKIα [42], synthetic oligonucleotides encoding the NES and NES(m) peptides were inserted between the *GFP *and *MTH1 *genes in the *GFP-MTH1 *construct [14]. Plasmids used in this study are listed in Table 1.

**Table 1:** Plasmids used in this study

Plasmid	Description	Source
GFP-Mth1	Mth1-GFP fusion protein (pUG34 or pUG36)	17
GFP-NES-Mth1	Mth1-GFP fusion protein with NES	This study
GFP-NES (m)-Mth1	Mth1-GFP fusion protein with NES (L changed to A)	This study
GFP-Grr1	Grr1-GFP fusion protein	This study
GFP-Grr1ΔN	Grr1-GFP fusion protein without amino acids from 1 to 280 [26]	This study
GFP-Yck1	Yck1-GFP fusion protein	This study
GFP-Yck2	Yck2-GFP fusion protein	This study
Mth1 ID 1	GFP-Mth1 fusion protein without amino acids from 1 to 20	This study
Mth1 ID 2	GFP-Mth1 fusion protein without amino acids from 81 to 90	This study
Mth1 ID 3	GFP-Mth1 fusion protein without amino acids from 88 to 119	This study
Mth1 ID 4	GFP-Mth1 fusion protein without amino acids from 118 to 138	This study
Mth1 ID 5	GFP-Mth1 fusion protein without amino acids from 137 to 157	This study
Mth1 ID 6	GFP-Mth1 fusion protein without amino acids from 156 to 180	This study
Mth1 ID 7	GFP-Mth1 fusion protein without amino acids from 179 to 188	This study
Mth1 ID 8	GFP-Mth1 fusion protein without amino acids from 187 to 200	This study
Mth1 ID 9	GFP-Mth1 fusion protein without amino acids from 198 to 214	This study
Mth1 ID 10	GFP-Mth1 fusion protein without amino acids from 213 to 236	This study
Mth1 ID 11	GFP-Mth1 fusion protein without amino acids from 235 to 262	This study
Mth1 ID 12	GFP-Mth1 fusion protein without amino acids from 261 to 299	This study
Mth1 ID 13	GFP-Mth1 fusion protein without amino acids from 298 to 319	This study
Mth1 ID 14	GFP-Mth1 fusion protein without amino acids from 318 to 327	This study
Mth1 ID 15	GFP-Mth1 fusion protein without amino acids from 326 to 343	This study
Mth1 ID 16	GFP-Mth1 fusion protein without amino acids from 342 to 363	This study
Mth1 ID 17	GFP-Mth1 fusion protein without amino acids from 363 to 380	This study
Mth1 ID 18	GFP-Mth1 fusion protein without amino acids from 390 to 433	This study
Mth1 ID 2 tag-less	Mth1 without amino acids from 81 to 90	This study
Mth1 ID 4 tag-less	Mth1 without amino acids from 118 to 138	This study
Mth1 ID 6 tag-less	Mth1 without amino acids from 156 to 180	This study
Mth1 ID 15 tag-less	Mth1 without amino acids from 326 to 343	This study

### Fluorescence microscopy and FRAP (Fluorescence Recovery After Photobleaching)

GFP-fusion proteins expressed in yeast cells were visualized using a Zeiss LSM 510 META confocal laser scanning microscope with a 63× Plan-Apochromat 1.4 NA Oil DIC objective lens (Zeiss) [17]. All images documenting GFP localization were acquired with the Zeiss LSM 510 software version 3.2. For FRAP of GFP-Mth1, one of the foci was bleached with a laser pulse and the subsequent recovery of fluorescence was monitored [17].

### Western blotting

Western blotting was performed as described previously [6]. Briefly, 5 ml of yeast cells (O.D_600 _= 1.2) were collected by centrifugation at 3,000 rpm in a table-top centrifuge for 5 min. The cell pellets were resuspended in 100 μl of SDS-buffer (50 mM Tris-HCl, pH 6.8, 10% glycerol, 2% SDS, 5% β-mercaptoethanol) and boiled for 5 min. After the lysates were cleared by centrifugation at 12,000 rpm for 10 min., soluble proteins were resolved by SDS-PAGE and transferred to PVDF membrane (Millipore). The membranes were incubated with appropriate antibodies in TBST buffer (10 mM Tris-HCl, pH 7.5, 150 mM NaCl, 0.05% Tween-20) and proteins were detected by the enhanced chemiluminescence (ECL) system (Pierce).

### β-galactosidase assay

To assay β-galactosidase activity with yeast cells expressing appropriate *lacZ *reporters, yeast cells were grown to mid-log phase and assay was performed as described previously [6]. Results were reported in Miller Units [(1,000 × OD_420_)/(*T *× *V *× OD_600_), where OD_420 _was the optical density at 420 nm, *T *was the incubation time in minutes, and *V *is the volume of cells in milliliters]. The reported enzyme activities were averages of results from triplicates of three different transformants.

## Authors' contributions

SP and SC were responsible for all the experimental work. JC conceived of the study and was involved in writing the manuscript. JK participated in study design and analyses and wrote the manuscript with input from SP. All authors have read and approved the final manuscript.

## References

[B1] Ozcan S Johnston M Function and regulation of yeast hexose transporters Microbiol Mol Biol Rev 1999 63 3 554 569 10477308 10.1128/mmbr.63.3.554-569.1999 PMC103746

[B2] Rolland F Winderickx J Thevelein JM Glucose-sensing and -signalling mechanisms in yeast FEMS Yeast Res 2002 2 2 183 201 12702307 10.1111/j.1567-1364.2002.tb00084.x

[B3] Johnston M Kim JH Glucose as a hormone: receptor-mediated glucose sensing in the yeast Saccharomyces cerevisiae Biochem Soc Trans 2005 33 Pt 1 247 252 15667318 10.1042/BST0330247

[B4] Ozcan S Johnston M Three different regulatory mechanisms enable yeast hexose transporter (HXT) genes to be induced by different levels of glucose Mol Cell Biol 1995 15 3 1564 1572 7862149 10.1128/mcb.15.3.1564 PMC230380

[B5] Ozcan S Johnston M Two different repressors collaborate to restrict expression of the yeast glucose transporter genes HXT2 and HXT4 to low levels of glucose Mol Cell Biol 1996 16 10 5536 5545 8816466 10.1128/mcb.16.10.5536 PMC231553

[B6] Kim JH Polish J Johnston M Specificity and regulation of DNA binding by the yeast glucose transporter gene repressor Rgt1 Mol Cell Biol 2003 23 15 5208 5216 10.1128/MCB.23.15.5208-5216.2003 12861007 PMC165726

[B7] Kim JH Immobilized DNA-binding assay, an approach for in vitro DNA-binding assay Anal Biochem 2004 334 2 401 402 10.1016/j.ab.2004.06.045 15494148

[B8] Polish JA Kim JH Johnston M How the Rgt1 transcription factor of Saccharomyces cerevisiae is regulated by glucose Genetics 2005 169 2 583 594 10.1534/genetics.104.034512 15489524 PMC1449106

[B9] Tomas-Cobos L Sanz P Active Snf1 protein kinase inhibits expression of the Saccharomyces cerevisiae HXT1 glucose transporter gene Biochem J 2002 368 Pt 2 657 663 10.1042/BJ20020984 12220226 PMC1223017

[B10] Lakshmanan J Mosley AL Ozcan S Repression of transcription by Rgt1 in the absence of glucose requires Std1 and Mth1 Curr Genet 2003 44 1 19 25 10.1007/s00294-003-0423-2 14508605

[B11] Mosley AL Lakshmanan J Aryal BK Ozcan S Glucose-mediated phosphorylation converts the transcription factor Rgt1 from a repressor to an activator J Biol Chem 2003 278 12 10322 10327 10.1074/jbc.M212802200 12527758

[B12] Flick KM Spielewoy N Kalashnikova TI Guaderrama M Zhu Q Chang HC Wittenberg C Grr1-dependent inactivation of Mth1 mediates glucose-induced dissociation of Rgt1 from HXT gene promoters Mol Biol Cell 2003 14 8 3230 3241 10.1091/mbc.E03-03-0135 12925759 PMC181563

[B13] Spielewoy N Flick K Kalashnikova TI Walker JR Wittenberg C Regulation and recognition of SCFGrr1 targets in the glucose and amino acid signaling pathways Mol Cell Biol 2004 24 20 8994 9005 10.1128/MCB.24.20.8994-9005.2004 15456873 PMC517892

[B14] Kim JH Brachet V Moriya H Johnston M Integration of transcriptional and posttranslational regulation in a glucose signal transduction pathway in Saccharomyces cerevisiae Eukaryot Cell 2006 5 1 167 173 10.1128/EC.5.1.167-173.2006 16400179 PMC1360249

[B15] Ozcan S Dover J Rosenwald AG Wolfl S Johnston M Two glucose transporters in Saccharomyces cerevisiae are glucose sensors that generate a signal for induction of gene expression Proc Natl Acad Sci USA 1996 93 22 12428 12432 10.1073/pnas.93.22.12428 8901598 PMC38008

[B16] Ozcan S Dover J Johnston M Glucose sensing and signaling by two glucose receptors in the yeast Saccharomyces cerevisiae Embo J 1998 17 9 2566 2573 10.1093/emboj/17.9.2566 9564039 PMC1170598

[B17] Pasula S Jouandot D Kim JH Biochemical evidence for glucose-independent induction of HXT expression in Saccharomyces cerevisiae FEBS Lett 2007 581 17 3230 3234 10.1016/j.febslet.2007.06.013 17586499 PMC2040036

[B18] Moriya H Johnston M Glucose sensing and signaling in Saccharomyces cerevisiae through the Rgt2 glucose sensor and casein kinase I Proc Natl Acad Sci USA 2004 101 6 1572 1577 10.1073/pnas.0305901101 14755054 PMC341776

[B19] Schmidt MC McCartney RR Zhang X Tillman TS Solimeo H Wolfl S Almonte C Watkins SC Std1 and Mth1 proteins interact with the glucose sensors to control glucose-regulated gene expression in Saccharomyces cerevisiae Mol Cell Biol 1999 19 7 4561 4571 10373505 10.1128/mcb.19.7.4561 PMC84254

[B20] Schulte F Wieczorke R Hollenberg CP Boles E The HTR1 gene is a dominant negative mutant allele of MTH1 and blocks Snf3- and Rgt2-dependent glucose signaling in yeast J Bacteriol 2000 182 2 540 542 10.1128/JB.182.2.540-542.2000 10629208 PMC94311

[B21] Lafuente MJ Gancedo C Jauniaux JC Gancedo JM Mth1 receives the signal given by the glucose sensors Snf3 and Rgt2 in Saccharomyces cerevisiae Mol Microbiol 2000 35 1 161 172 10.1046/j.1365-2958.2000.01688.x 10632886

[B22] Kaniak A Xue Z Macool D Kim JH Johnston M Regulatory network connecting two glucose signal transduction pathways in Saccharomyces cerevisiae Eukaryot Cell 2004 3 1 221 231 10.1128/EC.3.1.221-231.2004 14871952 PMC329515

[B23] Ozcan S Freidel K Leuker A Ciriacy M Glucose uptake and catabolite repression in dominant HTR1 mutants of Saccharomyces cerevisiae J Bacteriol 1993 175 17 5520 5528 8366037 10.1128/jb.175.17.5520-5528.1993 PMC206608

[B24] Stochaj U Osborne M Kurihara T Silver P A yeast protein that binds nuclear localization signals: purification localization, and antibody inhibition of binding activity J Cell Biol 1991 113 6 1243 1254 10.1083/jcb.113.6.1243 2045410 PMC2289025

[B25] Blondel M Bach S Bamps S Dobbelaere J Wiget P Longaretti C Barral Y Meijer L Peter M Degradation of Hof1 by SCF(Grr1) is important for actomyosin contraction during cytokinesis in yeast Embo J 2005 24 7 1440 1452 10.1038/sj.emboj.7600627 15775961 PMC1142548

[B26] Li FN Johnston M Grr1 of Saccharomyces cerevisiae is connected to the ubiquitin proteolysis machinery through Skp1: coupling glucose sensing to gene expression and the cell cycle Embo J 1997 16 18 5629 5638 10.1093/emboj/16.18.5629 9312022 PMC1170195

[B27] Feng Y Davis NG Akr1p and the type I casein kinases act prior to the ubiquitination step of yeast endocytosis: Akr1p is required for kinase localization to the plasma membrane Mol Cell Biol 2000 20 14 5350 5359 10.1128/MCB.20.14.5350-5359.2000 10866691 PMC85984

[B28] Roth AF Feng Y Chen L Davis NG The yeast DHHC cysteine-rich domain protein Akr1p is a palmitoyl transferase J Cell Biol 2002 159 1 23 28 10.1083/jcb.200206120 12370247 PMC2173492

[B29] Babu P Deschenes RJ Robinson LC Akr1p-dependent palmitoylation of Yck2p yeast casein kinase 1 is necessary and sufficient for plasma membrane targeting J Biol Chem 2004 279 26 27138 27147 10.1074/jbc.M403071200 15105419

[B30] Hicke L Zanolari B Riezman H Cytoplasmic tail phosphorylation of the alpha-factor receptor is required for its ubiquitination and internalization J Cell Biol 1998 141 2 349 358 10.1083/jcb.141.2.349 9548714 PMC2148449

[B31] Marchal C Haguenauer-Tsapis R Urban-Grimal D A PEST-like sequence mediates phosphorylation and efficient ubiquitination of yeast uracil permease Mol Cell Biol 1998 18 1 314 321 9418878 10.1128/mcb.18.1.314 PMC121498

[B32] Gadura N Robinson LC Michels CA Glc7-Reg1 phosphatase signals to Yck1,2 casein kinase 1 to regulate transport activity and glucose-induced inactivation of Saccharomyces maltose permease Genetics 2006 172 3 1427 1439 10.1534/genetics.105.051698 16361229 PMC1456300

[B33] Decottignies A Owsianik G Ghislain M Casein kinase I-dependent phosphorylation and stability of the yeast multidrug transporter Pdr5p J Biol Chem 1999 274 52 37139 37146 10.1074/jbc.274.52.37139 10601275

[B34] Estrada E Agostinis P Vandenheede JR Goris J Merlevede W Francois J Goffeau A Ghislain M Phosphorylation of yeast plasma membrane H+-ATPase by casein kinase I J Biol Chem 1996 271 50 32064 32072 10.1074/jbc.271.50.32064 8943257

[B35] Klasson H Fink GR Ljungdahl PO Ssy1p and Ptr3p are plasma membrane components of a yeast system that senses extracellular amino acids Mol Cell Biol 1999 19 8 5405 5416 10409731 10.1128/mcb.19.8.5405 PMC84383

[B36] Andreasson C Ljungdahl PO Receptor-mediated endoproteolytic activation of two transcription factors in yeast Genes Dev 2002 16 24 3158 3172 10.1101/gad.239202 12502738 PMC187503

[B37] Abdel-Sater F El Bakkoury M Urrestarazu A Vissers S Andre B Amino acid signaling in yeast: casein kinase I and the Ssy5 endoprotease are key determinants of endoproteolytic activation of the membrane-bound Stp1 transcription factor Mol Cell Biol 2004 24 22 9771 9785 10.1128/MCB.24.22.9771-9785.2004 15509782 PMC525479

[B38] Gaber RF Ottow K Andersen HA Kielland-Brandt MC Constitutive and hyperresponsive signaling by mutant forms of Saccharomyces cerevisiae amino acid sensor Ssy1 Eukaryot Cell 2003 2 5 922 929 10.1128/EC.2.5.922-929.2003 14555474 PMC219377

[B39] Liu Z Thornton J Spirek M Butow RA Activation of the SPS amino acid-sensing pathway in Saccharomyces cerevisiae correlates with the phosphorylation state of a sensor component, Ptr3 Mol Cell Biol 2008 28 2 551 563 10.1128/MCB.00929-07 17984223 PMC2223413

[B40] Goldstein AL Pan X McCusker JH Heterologous URA3MX cassettes for gene replacement in Saccharomyces cerevisiae Yeast 1999 15 6 507 511 10.1002/(SICI)1097-0061(199904)15:6<507::AID-YEA369>3.0.CO;2-P 10234788

[B41] Ma H Kunes S Schatz PJ Botstein D Plasmid construction by homologous recombination in yeast Gene 1987 58 2-3 201 216 10.1016/0378-1119(87)90376-3 2828185

[B42] Feng Y Davis NG Akr1p and the type I casein kinases act prior to the ubiquitination step of yeast endocytosis: Akr1p is required for kinase localization to the plasma membrane Mol Cell Biol 2000 20 14 5350 5359 10.1128/MCB.20.14.5350-5359.2000 10866691 PMC85984

